# Short and long term effect of treatment non-pharmacological and lifestyle in patients with metabolic syndrome

**DOI:** 10.1186/s13098-020-0522-y

**Published:** 2020-02-14

**Authors:** Ana Denise Brandão, Jeferson Hernandes da Silva, Sarah Mariane Oliveira Lima, Leiciane Lima, Bhianca Loize, Antônio Adolfo Mattos de Castro, Claudia Kümpel, Elias Ferreira Porto

**Affiliations:** 1 Adventist University of São Paulo (UNASP), Estrada de Itapecerica da Serra 5859, São Paulo City, São Paulo Province Zip Code 5858001 Brazil; 2 Federal University of Pampa (UNIPAMPA), Uruguaiana, Brazil

**Keywords:** Metabolic syndrome, Cardiac rehabilitation, Life style

## Abstract

**Background:**

Metabolic syndrome (MS) is a complex disorder represented by a set of cardiovascular risk factors usually related to central fat deposition, insulin resistance, hypertension and dyslipidemia. It is associated with accelerated atherosclerosis in response to chronic inflammation and vascular endothelial dysfunction, increasing overall mortality. The objective to evaluate the short and long term effect of the comprehensive cardiac rehabilitation program and intensive lifestyle intervention in metabolic syndrome patients.

**Methods:**

This is longitudinal interventional study. All patients underwent a 20-session cardiac rehabilitation program with aerobic and resisted exercises as well as an educational program for lifestyle changes and follow up 1 year.

**Results:**

Forty seven patients participated in the present study, but only 28 concluded the follow up. 77.7% were females and the majority was older than 60 years (63.1%). After cardiac rehabilitation, the percentage of overweight (0.04) patients who controlled the SBP (0.04) increased, and obesity levels I and II were reduced, as well as a significant reduction in total cholesterol and triglycerides (0.01 and 0.05), all of these variables remained similar after 1 year of follow-up. After cardiac rehabilitation all participants were practicing the five factors of healthy lifestyle, and reduced to 73% after follow up.

**Conclusion:**

A comprehensive cardiac rehabilitation program and lifestyle change is an effective approach in the treatment of patients with MS mainly, it has positive short and long term effects on weight control, reducing total cholesterol and triglycerides.

## Background

Metabolic Syndrome (MS) is a complex disorder represented by a set of cardiovascular risk factors usually related to central fat deposition, insulin resistance, hypertension and dyslipidemia [1]. It is associated with cardiovascular disease increasing overall and cardiovascular mortality up to two- and three-fold, respectively. Studies have shown that the prevalence of metabolic syndrome is high and varies with age [2]. The unadjusted prevalence of metabolic syndrome in the study population was 30.1% (CI 95% 29.2–31.0) and age-standardized prevalence was 33.7% (CI 95% 32.8–34.6). The prevalence increased with age in both sexes. The metabolic syndrome was more commonly seen in women than in men (42% vs. 24%, p < 0.001) [2]. Low HDL-C was the most common metabolic abnormality in both sexes. In this study it was seen that most of those with metabolic syndrome had three components of the syndrome (58%), 33% had four, and 9% had five components [3].

Health-related lifestyle can be a very effective strategy that is being used mainly in the prevention of chronic degenerative diseases [4]. Some studies have already shown positive associations between health-related lifestyle and components of metabolic syndrome improvement: systemic arterial blood, hypertriglyceridemia, high blood glucose and HDL-c reductions [5, 6] in a study followed for a mean of 3.2 years after random assignment to intensive lifestyle intervention, metformin therapy, or placebo the incidence of the metabolic syndrome was reduced by 41% in the lifestyle group (p < 0.001) and by 17% in the metformin group (p = 0.03) compared with placebo [6]. In addition, cardiac rehabilitation programs are considered good strategies for overall survival; it includes physical training, eating habits reeducation, smoke cessation and stress management [7].

Adopting a intensive lifestyle intervention associated to a cardiopulmonary rehabilitation program may be an effective low-cost alternative to reduce cardiovascular diseases risks, fatigue and loss of functional capacity associated to metabolic syndrome to short and long term [6–8]. In view of this, the objective of the study to evaluate the short and long term effect of the comprehensive cardiac rehabilitation program and intensive lifestyle intervention in metabolic syndrome patients.

## Materials and methods

This is a longitudinal study conducted in a consecutive sample 67 patients with metabolic syndrome, at the Rehabilitation Center of the University Polyclinic (Adventist University of São Paulo—UNASP, Brazil). In order to elaborate the guiding question of our research, we used the PICO [9, 10] strategy, acronym described below:

- P (patient or population): it is related to the description of the population under investigation, or even, definition of the condition of interest (individuals with metabolic syndrome);
- I (Intervention or exposure): refers to the description of what will be done with the population or patients (cardiac rehabilitation program);
- C (comparison or control): refers to the description of the criteria that will be used to evaluate the effectiveness of the intervention (pre-post intervention);
- O (outcome): corresponds to the description of the outcome of interest (cardiovascular risk; functional capacity; fatigue).

The Research Ethics Committee of UNASP approved the research under the number 2.170.175, in agreement with Resolution No. 466/2012 of the National Health Council and Declaration of Helsinki (WMA, 2013) [11].

The sample of this study consisted of 67 patients diagnosed with metabolic syndrome and were invited to participate in the Cardiac Rehabilitation Program (CRP). Both genders participated in the study who presented minimally three of the five criteria for metabolic syndrome and were older than 40 years old. Patients with metabolic syndrome who were undergoing chemotherapy, with hemodynamic instability and uncontrolled arrhythmias were excluded.

To evaluate cardiovascular risk, the Framingham Questionnaire was used, total and fractioned cholesterol, triglycerides, fasting blood glucose, blood pressure, weight, body mass index (BMI) and abdominal circumference, presence of diabetes, smoking habit. All participants were strongly encouraged to practice five healthy lifestyle habits (no smoking, no alcohol, physical activity 3–5 times a week, avoid foods, processed, embedded and high in fat, and sleep between 7 and 8 h a day), to assess this condition participant answered a questionnaire containing questions about these habits. The global fatigue was evaluate by the Chalder’s Fatigue Scale [13].

Also, patients performed an incremental lower limb test (BRUCE protocol) [14], a 6-min walk test and all patients accomplished a comprehensive cardiac rehabilitation program. All of these variables were baseline, post-rehabilitation and final follow-up measurements.

### Framingham questionnaire

Framingham score is an instrument used to calculate the risk of cardiovascular event. The following parameters are used: systolic blood pressure, total cholesterol, LDL-cholesterol, HDL-cholesterol, age, smoking and presence of diabetes. The Framingham score is 0 to 25 points, and the higher the score, the higher the cardiovascular risk.

### Chalder’s fatigue Scale

The Chalder Scale is made up of six questions to quantify the patient’s overall fatigue. The first two questions should be answered by all individuals, and the last four questions should be answered only by individuals who respond positively to the first two questions. Questions 1 to 4 are answered with a value of one or zero according to the intensity and frequency of fatigue.

### Blood test

To perform the tests of total and fractionated cholesterol, triglycerides, fasting glucose, we collected 20 ml of venous blood from the fasting individual.

### Body composition

BMI was classified according to criteria proposed by the world health organization: BMI < 18.5 kg/m^2^ was considered as low weight, BMI > 18.5 < 24.9 kg/m^2^ as eutrophic; BMI > 25 kg/m^2^ < 29.9 kg/m^2^ as overweight; BMI > 30 kg/m^2^ < 34.9 kg/m^2^ as obesity grade I; BMI > 35 kg/m^2^ < 39.9 kg/m^2^ as obesity grade II and BMI > 40 kg/m^2^ as obesity grade III [17].

### Metabolic syndrome criteria

The metabolic syndrome was defined by the presence of three or more of the following components: abdominal obesity, hypertriglyceridemia, low HDL-C, high blood pressure, and high fasting glucose.

### Cardiac rehabilitation program

Patients with metabolic syndrome performed a comprehensive rehabilitation program with 20 sessions, each lasting 60 min, 4 times per week. All patients were assessed by our team doctors and then referred to rehabilitation. Each session had 30 min of aerobic training for lower limbs on a treadmill. This training started with 3 min of warm-up, with 2.6 km/h of speed, 22 min at a fixed speed and treadmill inclination ranging from 60 to 80% intensity of the maximum value reached during the Bruce protocol; finally, the last 5 min had 2.7 km/h speed decreased for cool down purposes.

Upper limbs exercises comprises of 15 min of strengthening exercises and 15 min of lower limb strengthening exercise; 2 sets of 10 reps for each exercise (with dumbbells and ankle weights ranging from 1 to 3 kg, theraband and Swiss ball).Specific loads were given for each individual and an interval of 60 s between each exercise was provided.

### Life style habits

The participants were encouraged intensively to practice health-promoting practices, through classes given three times per week by our facilities’ physiotherapists, athletic trainers and nutritionists. Adoption of a diet rich of fruits and vegetables was oriented in order to meet metabolic targets [15] (mostly 3 to 5 times a day of fruit intake–the diet provided was comprehensive and, generally, oriented to all). No individual diet consult was provided, Physical activity practice for at least 30 min three to five times a week, breathing fresh air and frequent water intake, smoking cessation and avoidance of alcoholic beverages and soft drinks, taking to 7 to 8 h/day of sleeping, adequate exposure to sunlight and confidence in God [16] were also oriented.

### Follow up

At the end of the rehabilitation program all patients were invited to return to the exams after 12 months. Contact was sought with all patients, and invited to come to the polyclinics to perform the examinations as previously agreed, but only 28 performed all examinations at the end of 12 months.

### Statistical analysis

The Kolmogorov–Smirnov normality test was used to evaluate the distribution of data in relation to normality. When the data were normal, parametric tests were used for analysis. The data are presented in mean and standard deviation, the paired test was used to compare the differences between the anthropometric variables and the functional capacity before and after intervention between the groups. The proportions were compared with qui square teste. For proportions compared by the Chi square test, the odds ratio was used to assess the risk of loss of rehabilitation program effects during follow-up.

We used the Cohen d test to calculate the effect size; it was considered no effect range between 0.0 and 0.1, small effect range between 0.11 and 0.4, moderate effect range between 0.41 and 0.6 and great effect range greater than 0.6. It was considered p < 0.05 as statistical significant.

## Results

**Fig. 1:**
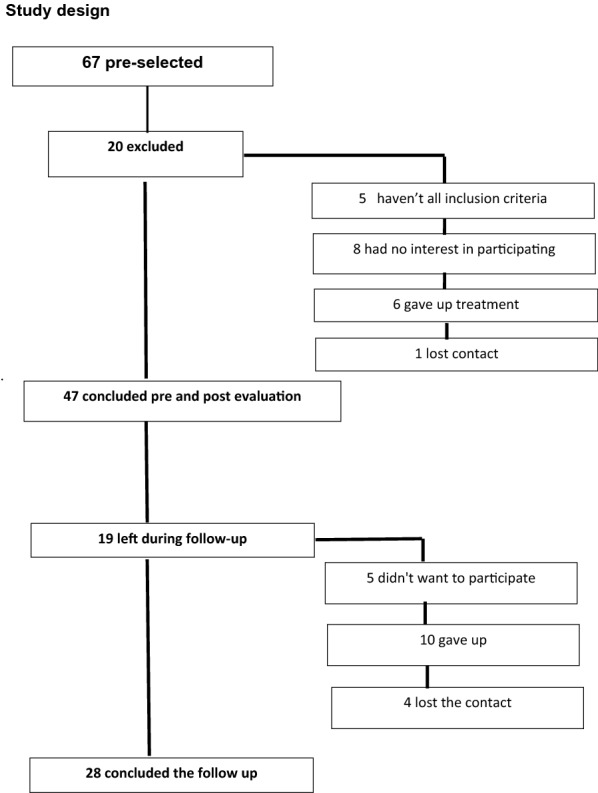
Study design

Initially the sample consisted of 67 patients with metabolic syndrome, but 20 were excluded 5 of them haven’t all inclusion criteria, 8 had no interest in participating, 6 gave up treatment and one we lost contact. Of these 47 completed an accomplished rehabilitation program and started follow-up, however 5 declined to continue, 10 started but dropped out and 4 patients lost contact with 28 patients remaining until the end of follow-up, Fig. 1.

The average age was 61 years, the vast majority were female, with body composition predominating obesity degree I and degree II, and after rehabilitation there was a change in body composition significantly reducing the proportion of individuals with obesity degree I and degree II, and increased proportion of overweight, during follow-up there was no difference in relation to post-rehabilitation. The proportion of subjects who maintained SBP controlled increased significantly and remained during follow-up. There was no significant increase in the proportion of subjects who improved control of diabetes seen by HBA1C, triglycerides, and waist circumference, Table 1.

**Table 1:** Demographic characteristics at baseline and after cardiac rehabilitation

	Pre-rehabilitation (%) (n = 47)	Post-rehabilitation (%) (n = 47)	p	Follow up (n = 28)	p
Man (%)	22.2	22.2	0.99	22.2	0.99
Woman (%)	77.8	77.8	0.98	77.8	0.98
Age (years)	61.6 ± 6.2	61.9 ± 6.2	0.54	62.6 ± 9.8	0.4
Overweight	22.2	33.3	*0.04*	33.3	0.99
Obesity degree I (%)	44.4	38.8	*0.049*	44.4	0.055
Obesity degree II (%)	22.2	11	*0.05*	5.5	0.058
Obesity degree III (%)	5.5	11	0.41	16.6	0.42
HBA1C < 6%	33.3	22.2	0.48	16.6	0,44
SBH controlled (%)	11	33.3	*0.04*	33.3	0.99
Total cholesterol > 200 mg/dl (%)	55.5	66.6	0.74	60	0.78
HDL cholesterol					
Man < 40 mg/dl (%)	75	50	*0.032*	25	*0.01*
Woman < 50 mg/dl (%)	64.2	71.4	0.65	71	0.99
LDL > 135 mg/dl (%)	77.7	83.3	0.87	90	0.78
Triglycerides > 150 mg/dl (%)	66.6	77.7	0.65	80	0.85
Waist circumference					
Woman < 88 cm (%)	0	0		25	
Man < 102 cm (%)	0	25		25	

The values highlighted in italics represent p < 0.05 pre-rehabilitation versus post-rehabilitation or post-rehabilitation versus follow up

*HDL* high density lipoprotein, *LDL* low density lipoprotein

Table 2 shows the results and physical capacity before and after rehabilitation and during follow-up, as well as blood pressure outcome. It can be seen that there was a reduction in overall fatigue after rehabilitation, however it returned to baseline during follow-up. There was an increase in SMWD (p = 0.038) and time in the Bruce protocol (p = 0.009) and a reduction in systolic blood pressure (p = 0.014), which were maintained during follow-up.

**Table 2:** Comparison of anthropometric results and physical capacity before and after intervention

	Pre-rehabilitation (%) (n = 47)	Post-rehabilitation (%) (n = 47)	p	Follow up (n = 28)	p
BMI (kg/m^2^)	32.6 ± 3.9	32.3 ± 4.7	0.41	32.4 ± 5.0	0.85
Waist circumference (cm)	108.8 ± 7.4	106.5 ± 8.5	0.2	106.1 ± 8.5	0.8
Global fatigue (score)	2.5 ± 1.3	0.66 ± 1.1	*0.0001*	1.6 ± 1.0	*0.01*
SMWT (m)	474.1 ± 80.6	523.1 ± 71.8	*0.038*	499.6 ± 83.7	0.25
Bruce protocol (min)	3.1 ± 1.2	4.1 ± 1.1	*0.009*	3.2 ± 1.1	*0025*
Systolic blood pressure (mmHg)	141.2 ± 11.2	130.9 ± 13.9	*0.014*	124.5 ± 33.1	0.21
diastolic blood pressure (mmHg)	86.7 ± 11.2	81.1 ± 9.1	0.064	72.7 ± 18.2	0.32

The values highlighted in italics represent p < 0.05 pre-rehabilitation versus post-rehabilitation or post-rehabilitation versus follow up

*BMI* body mass index, *SMWT* 6 min walking test, *SD* standard deviation

Table 3 shows the values for total and fractionated cholesterol, triglycerides fasting glucose and glycosylated hemoglobin, before and after rehabilitation and during follow-up. There was a significant reduction in fasting glucose level (p = 0.017), total cholesterol (p = 0.01) and Triglycerides (p = 0.05) and there was no difference during follow-up.

**Table 3:** Values for total and fractionated cholesterol, triglycerides fasting glucose and glycosylated hemoglobin, before and after PRC

	Pre-rehabilitation (%) (n = 47)	Post-rehabilitation (%)(n = 47)	p	Follow up (n = 28)	p
Fasting glucose (mg/dl)	141.1 ± 68.7	123.1 ± 37.5	*0.017*	134.3 ± 58.2	0.27
HBA1C (mg/dl)	7.0 ± 1.8	6.9 ± 1.4	0.42	7.6 ± 1.7	0.13
Total cholesterol (mg/dl)	209.7 ± 40.9	188.2 ± 35.8	*0.01*	193.8 ± 35.7	0.37
HDL cholesterol (mg/dl)	46.5 ± 10.9	44.5 ± 10.8	0.29	40.1 ± 10.8	0.16
LDL cholesterol (mg/dl)	119.5 ± 35.9	108.3 ± 32.3	0.18	104.2 ± 31.5	0.38
Triglycerides (mg/dl)	209.5 ± 109	190.1 ± 65.3	*0.05*	190.3 ± 60.4	0.49

The values highlighted in italics represent p < 0.05 pre-rehabilitation versus post-rehabilitation or post-rehabilitation versus follow up

*HBA1C* A1c glycosylated hemoglobin, *HDL* high density lipoprotein, *LDL* low density lipoprotein

Table 4 are absolute and relative frequencies to metabolic syndrome criteria, pre and post rehabilitation and follow up, and it can be observed that the proportion of five and four metabolic syndrome criteria decreased in post rehabilitation and increased during follow-up. For three criteria it was practically maintained after rehabilitation and reduction during follow up, and for two criteria it increased after rehabilitation and returned to the same baseline ratio.

**Table 4:** Absolute and relative frequencies to metabolic syndrome criteria, pre and post rehabilitation

Variables	Pre-rehabilitation [n (%)]	Post-rehabilitation [n (%)]	Follow up [n (%)]
Five metabolic syndrome criteria	11 (23.4)	0 (0)	5 (10.7)
Four metabolic syndrome criteria	16 (34)	10 (21.2)	11 (39.2)
Tree metabolic syndrome criteria	20 (42.5)	19 (40)	7 (25)
Two metabolic syndrome criteria	0 (0)	10 (21.2)	5 (17.8)
One metabolic syndrome criteria	0 (0)	7 (14.8)	0 (0)
Zero metabolic syndrome criteria	0 (0)	0 (0)	0 (0)
Total	47 (100)	47 (100)	28 (100)

Table 5 shows the effect size for some variables that showed a statistically improvement between pre and post rehabilitation. As for total cholesterol, LDL cholesterol and distal measurement of blood pressure we found to have moderate effect; nevertheless, we found a larger effect size as for triglycerides and systolic blood pressure. During follow-up the effect size was also analyzed in relation to the patient’s baseline, there was a moderate loss of effect for HDL cholesterol and Hb1AC, however there was moderate preservation of effect of rehabilitation for total cholesterol, LDL cholesterol, triglycerides, systolic and diastolic blood pressure.

**Table 5:** Effect size at short and long term of cardiac rehabilitation program for patients with metabolic syndrome

Variables	Pre and post rehabilitation				Pre rehabilitation and follow up			
	d Conhen	CI 95%	TDE	Effect size	d Conhen	CI 95%	TDE	Effect size
Fast glucose	0.33	0.74 to 0.07	0.45	Small effect	0.05	− 0.41 to 0.67	0.10	No effect
Hb1AC	0.11	0.29 to 0.51	0.53	Small effect	− 0.33	− 0.71 to 0.05	0.16	Moderate effect
Total cholesterol	0.52	0.10 to 0.90	0.64	Moderate effect	0.39	0.003 to 0.71	0.20	Moderate effect
HDL cholesterol	0.05	0.35 to 0.45	0.51	No effect	− 0.54	− 0.93 to − 0.14	0.28	Moderate effect
LDL cholesterol	0.5	0.08 to 0.91	0.63	Moderate effect	0.42	0.03 to 0.81	0.22	Moderate effect
Triglycerides	1.0	0.5 to 1.4	0.76	Great effect	0.54	0.14 to 0.95	0.13	Moderate effect
Systolic blood pressure	0.81	0.38 to 1.2	0.71	Great effect	0.51	0.1 to 0.9	0.32	Moderate effect
Diastolic blood pressure	0.61	0.17 to 1.1	0.66	Moderate effect	0.77	0.34 to 1.34	0.42	Moderate effect

*HBA1C* A1c glycosylated hemoglobin, *HDL* high density lipoprotein, *LDL* low density lipoprotein

As for the lifestyle was intensively stimulated so that the participants acquired five healthy habits, initially 23.3% already practiced these lifestyle habits, after rehabilitation 100% were practicing the five healthy lifestyle habits, however at the end of the follow-up only 44% were practicing all healthy lifestyle habits, and physical activity and healthy nutrition were the main habits abandoned following.

## Discussion

The effect of an accomplished rehabilitation in patients with metabolic syndrome is an important and obligatory evaluation due to the great impact of metabolic and physical changes present over their quality of life, functionality, morbidity and mortality of these patients.

The main results of this study were that patients with metabolic syndrome. First one, there was a reduction in the proportion of patients with obesity degree I and II, a significant increase in the proportion of patients who control SBP in the short and long term (1-year follow-up). Second one, was also found a positive effect of cardiac rehabilitation in short and long-term on PAS for fasting glucose, total cholesterol, triglycerides (1-year follow-up), in addition to the physical improvement seen by SMWD and Bruce protocol time. Third one, the effect size remained moderate after 1 year of follow-up for total cholesterol, triglycerides, LDL cholesterol, systolic and diastolic blood pressure. However, there was no effect on HDL cholesterol and waist circumference.

The reductions in the overall weight in this study are similar to what has been observed by other authors [8–10], however weight loss remained a year after the end of the rehab program, what is a new finding, it may have occurred because about 66% of patients continued their healthy lifestyle after the rehab program, Despite the continued to practice the five healthy living habits group presented more loss weight than did not continue to the five healthy living habits group, there was no statistically significant difference.

The importance of exercise in weight loss and prevention of weight regain is well accepted [11]. In general, exercise during weight loss appears to target loss of fat mass while preserving lean mass. The effects of exercise on total body weight are variable, but generally proportional to the accumulated total energy expenditure [12, 13]. Variables including duration of activity per day, frequency of activity per week, and intensity ultimately determine energy expenditure and the potential for weight loss. This reinforces the idea that constant exercise may preserve and increase muscle mass and supports the indication of rehabilitation for all metabolic syndrome patients, especially for the ones are obese.

Some authors report that the exercise can reduce total and fractionated cholesterol as well as contribute to blood pressure control [14, 15] Similar to the results found in this study. Most studies have shown beneficial changes in the levels and chemical composition of fractions and subfractions of HDL-cholesterol (HDL2-cholesterol, the main anti-atherogenic subfraction and HDL3-cholesterol) and LDL-cholesterol (small and dense LDL-cholesterol transformation considered more atherogenic, in large and less dense), after a program of aerobic exercises with different intensities, durations and frequencies, performed by individuals of different age groups and levels of cardiorespiratory fitness. Few were those who did not find significant changes in HDL-cholesterol and LDL-cholesterol levels with aerobic exercise [16, 17].

In a systematic review on the effects of physical rehabilitation on the relationship between HDL-cholesterol, LDL-cholesterol changes and aerobic training seems to be well defined. The acute or chronic effect of aerobic exercise, both low and high intensity and duration, can improve lipoprotein profile, stimulating the better functioning of enzymatic processes involved in lipid metabolism (increased lipoprotein lipase and lecithin cholesterol acyl transferase; decrease in hepatic lipase), favoring, mainly, increases in HDL-cholesterol and HDL2-cholesterol subfraction, as well as modifying the chemical composition of LDL-cholesterol, making them less atherogenic [18]. These may be the mechanisms by which patients with metabolic syndrome improved their lipid profile during the rehabilitation program, and maintained during 1-year follow-up.

When performing exercise in this modality, some effects are known such as the increase of the shear stress mediated by the flow in the arteries walls that improves endothelial function. These mechanisms increase the synthesis and release of nitric oxide that leads to endothelium vasodilation and inhibits multiple processes involving atherogenesis and thrombosis [19].

The mechanism for blood pressure reduction is already well described both in the short and long time; in the short time it is due to the release of pro-vasodilation factors by the vascular endothelium after exercise with moderate high intensity load. In a long time it occurs by vascular neo-formation in the muscles during anaerobiosis. Reductions of only 2 mmHg in diastolic blood pressure can substantially reduce the risk of diseases and deaths associated with hypertension [20].

One of the current recommendations in the scientific world is that effect sizes should be presented associated with levels of statistical significance. Since the p-values resulting from the statistical test results do not inform on the magnitude or importance of a difference, then the effect sizes (TDE) should be reported. In fact, TDEs give meaning to statistical tests, emphasize the power of statistical tests, reduce the risk of mere sample variation being interpreted as a real relation [21].

Despite the average reduction of 2 cm in the waist of our patients there was no statistically significant difference, similar to our results was found in another study, however they found statistical significance, but the reduction of the waist was 2 cm (102.1 ± 7, 5 cm versus 100.8 ± 7.4 cm; p = 0.03) [22]. Even our sample being larger than these authors. In another study with 22 healthy individuals with a mean age of 40 ± 8 years were allocated to the groups: control (CO), training endurance (ET) and intermittent training (IT). The protocols lasted 12 weeks, three times per week; and intensities of 10% below and 20% above the anaerobic threshold, the authors found a reduction in abdominal [23].

The main difference found between these studies is the presence of patients with metabolic syndrome, it may be necessary to study a larger number of patients to better understand this fact.

This study brings important clinical applications mainly related to the fact that small lifestyle changes, mostly focused on physical activity, may provide improvements in the clinical aspect of the metabolic syndrome. The limitations of this study are related to the fact that we only measured the behavior of these variables after the cardiac rehabilitation program, not being able to measure the effects over a long time, as well as to follow other variables such as hospitalization and mortality. However, this cannot invalidate our results.

## Conclusion

A comprehensive cardiac rehabilitation program and lifestyle change is an effective approach in the treatment of patients with MS mainly, it has positive short and long term effects on weight control, reducing total cholesterol and triglycerides.

## Data Availability

All authors declare that data and any supporting material regarding this manuscript is available and it can be requested at any time.
